# Supplementary material for: Giant switchable non thermally-activated conduction in 180° domain walls in tetragonal Pb(Zr,Ti)O3
Source: Nat Commun. 2022 Nov 24;13:7239. doi: 10.1038/s41467-022-34777-6 (PMC9700693; doi:10.1038/s41467-022-34777-6)
Supplement: Supplementary file 1 — Supplementary Information [file 41467_2022_34777_MOESM1_ESM.pdf]

NATURE COMMUNICATIONS

Supplementary Information

Giant switchable non thermally-activated conduction in 180° domain walls in tetragonal Pb(Zr,Ti)O_3

Felix Risch[1], Yuri Tikhonov[2], Igor Lukyanchuk[2], Adrian M. Ionescu[1], Igor Stolichnov[1]*

[1] Nanoelectronic Devices Laboratory (NanoLab), EPFL, 1015 Lausanne, Switzerland
[2] Laboratory of Condensed Matter Physics, University of Picardie, 80039 Amiens, France

*The correspondence should be sent to igor.stolitchnov@epfl.ch

List of Supplementary information:

Supplementary Table 1 | Comparison of domain wall conduction values in literature

Supplementary Figure 1 | Phase-loop & asymmetric IV characteristics of 90°- and 180°-DWs

Supplementary Figure 2 | Consecutive IV characteristics of 180°-DWs

Supplementary Figure 3 | Non-thermally domain wall conduction

Supplementary Figure 4 | Pristine 90°-DW conduction at cryogenic temperatures

Supplementary Figure 5 | Creation of surface particles after poling with high electric fields

Supplementary Note 1 | Fowler-Nordheim formalism and fitting parameters

Supplementary Table 1 | Comparison of domain wall conductance values in literature. In the table (next page), DW conductance values are compared together with the respective readout bias for different materials and DW configurations. Necessary writing/erasing biases to create the (charged) DWs are given as well as the used readout method and other remarks. Notably, significant conduction (>1nA) is observed only for strongly charged DWs prepared using a special poling procedure or formed transiently under voltage.

Supplementary Table 1 | Comparison of domain wall conductance values in literature

ref	Material	DW type, DW creation	Write/Erase Bias	Read Current	Readout method and critical dimension	Other remarks
1	$BiFeO_3$	lateral 71°-charged DWs (cDWs), which can be switched between a H-H and T-T configuration	switching between ±4V and ±6V for different device dimensions	15nA at 2.5V (for smallest device)	readout between tip and circular electrode connected by 4 cDWs (varying tip-electrode distance between 100-250nm)	negative current-temperature dependency shows non thermally activated conduction
2	$Pb(Zr_{0.2},Ti_{0.8})O_3$	vertical H-T 180°-DWs, created by polarization switching of c-domains	>2.5V	80pA at 2.25V	conduction measurements are carried out in UHV with AFM-tip as top electrode (70nm film thickness)	current-temperature measurements yield thermally activated conduction properties
3	$Pb(Zr_{0.1},Ti_{0.9})O_3$	vertical H-T 45°-DWs, as grown seperating ferroelastic a-domains from c-domains	as grown	40pA at 7.25V	conduction readout through tip at 4K within UHV-AFM setup (60nm film thickness)	weak current-temperature dependency suggests non thermally activated conduction
4	$BiFeO_3$	lateral H-H 71°-cDW, created by lateral polarization switching	2.3V-4V for different device dimensions	4nA at 4V; (for 3-terminal device 14nA at 5V)	conduction readout by 2(3)-terminal device structure between planar top electrodes (electrode distances between 50-200nm)	endurance and retention behaviour measurements showed low fatigue and good stability
5	$BiFeO_3$	lateral H-H 71°-cDW, created by lateral polarization switching	+8V	up to 1nA at 2V (for smallest device)	conduction measured either from cross-section of cAFM images or in a planar device configuration (electrode distances: 90-400nm)	larger device dimensions lead to straightening of the H-H DWs together with a decrease in conductivity
6	$LiNbO_3$	vertical H-H cDWs with varying angle due to cone-like domain nucleation	V_c=32V/-8V; subcoercive field tuning (4V/-1V) is possible after creation	30pA at 4V (single DW) & 1nA at 4V (device readout)	single DW readout by AFM-tip (crystal thickness ~500nm); device readout of multiple DWs using top electrode (size ~5um)	demonstration of memristive properties; increase of conductance by pulse trains through manipulation of DW angle
7	$ErMnO_3$	gradual varying H-H and T-T DW configurations in pristine crystal	as grown	<10pA at 3.5V	conduction measured through AFM tip acting as a top electrode (sample thickness ~200um)	continuous conduction variation with over one order of magnitude by changing DW orientation between H-H and T-T DWs
8	$BiFeO_3$	vertical H-H 71°-cDW, created under electric field	V_c=4.5V/-1.5V	100nA at 1.5V	DW creation and conduction readout in situ within the TEM experiment by a tungsten surface probe (20nm film thickness)	cDWs can only be created at positions in which there exists a 180°/109° domain junction
9	$(Bi_{0.9}La_{0.1})FeO_3$	vertical H-H cDWs, created by trailing field, i.e. poling with a lateral polarization preference	V_c=5V/-6V	2nA at 3V	conduction measured either as cross-section analysis of cAFM images or with the AFM-tip as the top electrode (45nm film thickness).	negative current-temperature dependency shows non thermally activated conduction
10	$BaTiO_3$	vertical 90° H-H cDW, created by frustrated poling during cooling over the phase-transition	as grown during phase-transition	50pA at 25V	conduction measured through 200um big top electrodes (crystal thickness ~200um)	negative current-temperature dependency shows non thermally activated conduction

Supplementary Figure 1 | Phase-loop & asymmetric IV characteristics of 90°- and 180°-DWs. a, IV curve measured with the AFM-tip as the top electrode showing the asymmetry in the IV characteristics of the 90°-DW. This behaviour can be attributed to the transport limited by the tip/sample interface. **b,** phase-loop of the piezoelectric response measured with the tip as a top electrode. As clearly seen, the loop is shifted by around 2.8V, resulting in coercive field values of ~+5V in the positive range and ~-1V in the negative one. **c**, IV curve measured with the AFM-tip as the top electrode showing the asymmetry in the IV characteristics similar to **a**. It is important to note that probing the 180°-DWs, which are created through polarization reversal, in the negative voltage range is only applicable in a very narrow range due to the asymmetric phase-loop. The coercive voltage on the negative side is around -1V. By applying a stronger negative bias, the polarized domain is modified and the DW is pushed away from its original position, making probing of its conductive properties impossible. This behaviour is actually used as an advantage in our configuration, the asymmetric phase-loops opens up the possibility to use the DW conduction on the positive side in a wider range up to ~+4V without destroying or moving the DW.

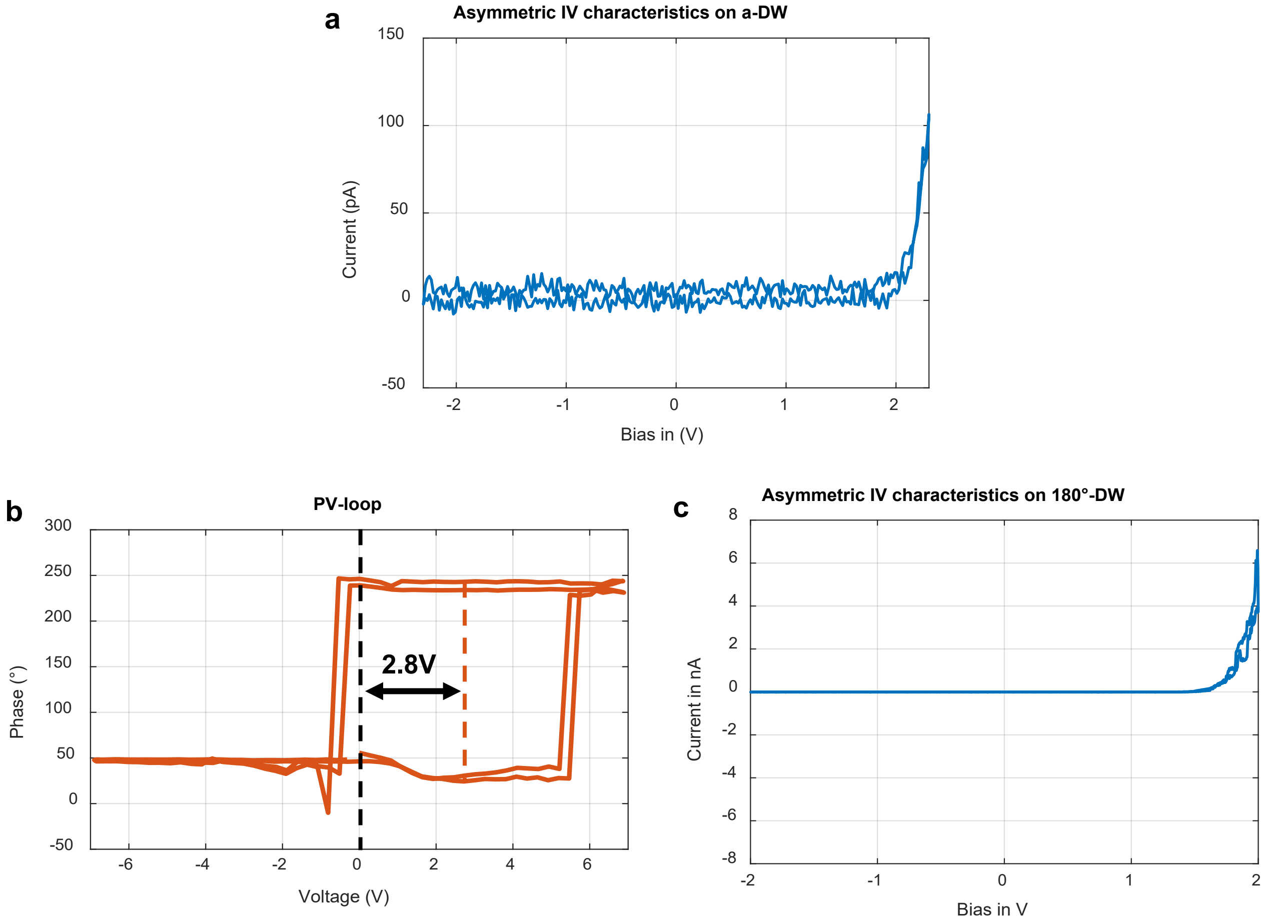

Supplementary Figure 2 | Consecutive IV characteristics of 180°-DWs. A series of 8 consecutive IV curves were performed on an artificially created 180°-DW using the AFM-tip as a top electrode. Clearly, the current readout is not destroying the conductivity measured through the DW nor displacing the DW to an extend that the conduction significantly drops. This confirms that the measured conduction is not associated with the volatile polarization switching currents. Because in the case of polarization switching current, the measured conduction would be associated with the displacement of the DW and would drastically decrease once the DW is pushed away.

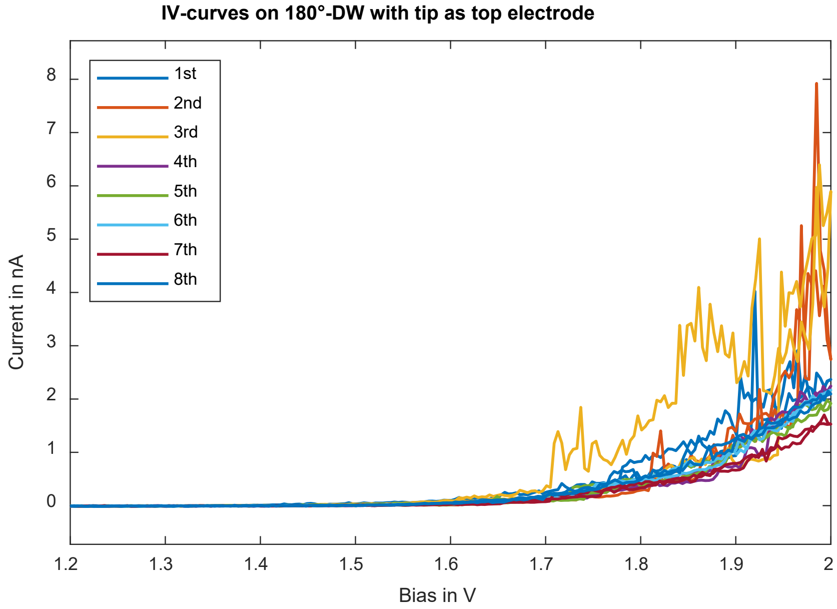

Supplementary Figure 3 | Non-thermally domain wall conduction. Temperature dependent measurements of the 180°-DW conductance have been carried out within the temperature range of 35-100°C. A ~700nm big domain has been created using the same procedure as in Fig. 1. **a,** cAFM scans with 1.7V sample bias have been taken at different temperatures of 100°C, 60°C and 35°C. **b,** the conduction along the DW path was averaged along a 5 point thick cross section line perpendicular to the path that follows the domain boundary (drawn path shown in the insert). A clear non-thermally activated behaviour is observed. The weak temperature dependence can partially be attributed to a polarization decrease and an increased effective tunnel barrier thickness at higher temperatures.

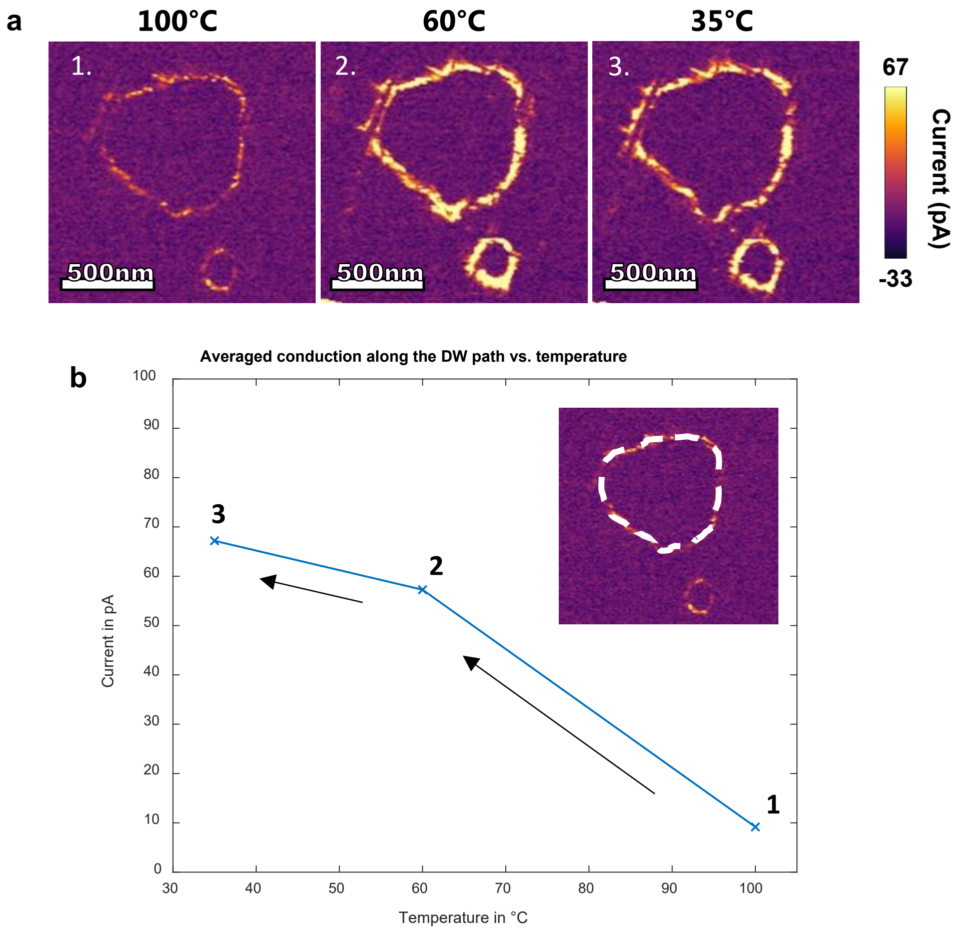

Supplementary Figure 4 | Pristine 90°-DW conduction at cryogenic temperatures. a, cAFM image at 42 K. The sample bias was 5.4V and current traces of several tens of pA were observed. **b,** cAFM image at 4 K. The sample bias was 6V and similar current values were observed in comparison to **a**. **c,** IV curves measured on a pristine 90°-DW at 4K. As already seen in the cAFM scans, a weak tens of pA conduction is maintained even at very low temperatures.

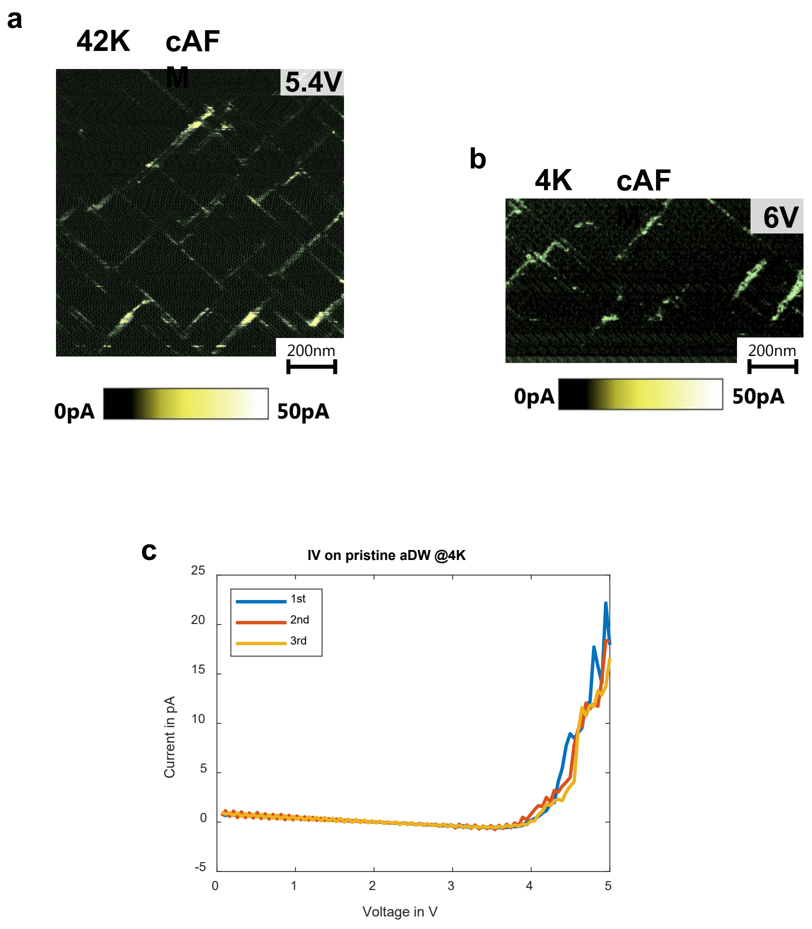

Supplementary Figure 5 | Creation of surface particles after poling with high electric fields. Before and after topography images of the studied area in Fig. 3a-d. The effect of using a high electric-field for poling (>7V) is observed in the formation of surface particles. The PFM-phase images serve to visualize the area, which is affected by the high electric fields. These particles remained even after scanning in contact mode. By using lower electric field, fewer particles could be produced but at the same time, the uniformity of the poling is reduced.

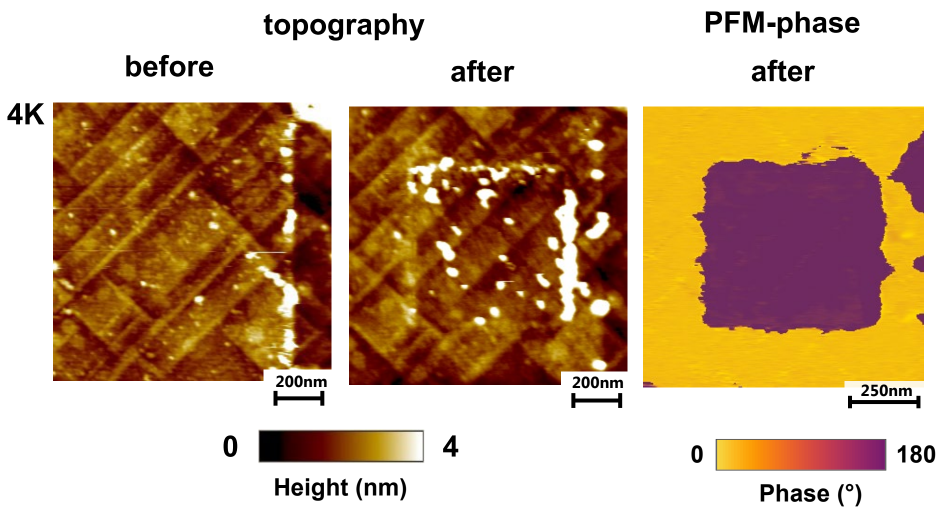

Supplementary Note 1 | Fowler-Nordheim formalism and fitting parameters The I-V curve in Fig. 1o was fitted using classic Fowler-Nordheim formula which describes tunneling through the triangular potential barrier ϕ as a function of applied electric field *F:*

$$I = S\frac{e^3 m_{tip}}{8\pi h m_{PZT}\phi} \times F^2 exp\left(-\frac{8\pi\sqrt{2m_{PZT}}}{3he}\frac{\phi^{3/2}}{F}\right), \quad (1)$$

where m_{tip} and m_{PZT} are electron effective tunneling masses for the tip and PZT. S, *e*, and *h* are the effective tunneling area, electron charge and Plank constant, respectively. In order to convert the applied voltage *V* to the electric field *F* we used a model of spherical tip of radius R[11], which yields a convenient approximation applicable within the relevant length scale: *F=V/R*. The fit in Fig.1o was obtained for the potential barrier $\phi = 0.8eV$, $m_{tip} = m_e$,$m_{PZT} = 3m_e$ (where m_e is the free electron mass), effective tip radius of 2nm and *S*=10nm^2.

Supplementary References:

1. Yang, W. *et al.* Nonvolatile Ferroelectric-Domain-Wall Memory Embedded in a Complex Topological Domain Structure. *Advanced Materials* **34**, 2107711 (2022).
2. Guyonnet, J. *et al.* Conduction at domain walls in insulating $Pb(Zr_{0.2}Ti_{0.8})O_3$ thin films. *Advanced Materials* **23**, 5377–5382 (2011).
3. Stolichnov, I. *et al.* Bent ferroelectric domain walls as reconfigurable metallic-like channels. *Nano Letters* **15**, 8049-8055 (2015).
4. Jiang, J. *et al.* Temporary formation of highly conducting domain walls for non-destructive read-out of ferroelectric domain-wall resistance switching memories. *Nature materials* **17**, 49-56 (2018).
5. Sharma, P. *et al.* Nonvolatile ferroelectric domain wall memory. *Science advances* **3**, e1700512 (2017).
6. Lu, H. *et al.* Electrical tunability of domain wall conductivity in $LiNbO_3$ thin films. *Advanced Materials* **31**, 1902890 (2019).
7. Meier, D. *et al.* Anisotropic conductance at improper ferroelectric domain walls. *Nature materials* **11**, 284-288 (2012).
8. Li, L. *et al.* Giant resistive switching via control of ferroelectric charged domain walls. *Advanced Materials* **28**, 6574-6580 (2016).
9. Crassous, A. *et al.* Polarization charge as a reconfigurable quasi-dopant in ferroelectric thin films. *Nature nanotechnology* **10**, 614-618 (2015).
10. Sluka, T. *et al.* Free-electron gas at charged domain walls in insulating $BaTiO_3$. *Nature communications* **4**, 1808 (2013).
11. Molotskii, M. Generation of ferroelectric domains in atomic force microscope. *Journal of applied physics* **93**, 6234-6237 (2003).
